# Preparation and Electrical Properties of La_0.9_Sr_0.1_TiO_3+δ_

**DOI:** 10.3390/ma8031176

**Published:** 2015-03-17

**Authors:** Wenzhi Li, Zhuang Ma, Lihong Gao, Fuchi Wang

**Affiliations:** 1 School of Material Science and Engineering, Beijing Institute of Technology, Beijing 100081, China; E-Mails: liwenzhi0418@163.com (W.L.); hstrong929@bit.edu.cn (Z.M.); wangfuchi@bit.edu.cn (F.W.); 2 National Key Laboratory of Science and Technology on Materials under Shock and Impact, Beijing 100081, China

**Keywords:** La_0.9_Sr_0.1_TiO_3+δ_, dielectric properties, conductivity, band gap

## Abstract

La_1__−*x*_Sr*_x_*TiO_3+δ_ (LST) has been studied in many fields, especially in the field of microelectronics due to its excellent electrical performance. Our previous theoretical simulated work has suggested that LST has good dielectric properties, but there are rare reports about this, especially experimental reports. In this paper, LST was prepared using a solid-state reaction method. The X-rays diffraction (XRD), scanning electron microscope (SEM), broadband dielectric spectroscopy, impedance spectroscopy and photoconductive measurement were used to characterize the sample. The results show that the values of dielectric parameters (the relative dielectric constant ε_r_ and dielectric loss tanδ), dependent on temperature, are stable under 350 °C and the value of the relative dielectric constant and dielectric loss are about 52–88 and 6.5 × 10^−3^, respectively. Its value of conductivity increases with rise in temperature, which suggests its negative temperature coefficient of the resistance. In addition, the band gap of LST is about 3.39 eV, so it belongs to a kind of wide-band-gap semiconductor materials. All these indicate that LST has anti-interference ability and good dielectric properties. It could have potential applications as an electronic material.

## 1. Introduction

Ceramic materials have received great attention because of their good stability, heat resistance, component of flexibility and many other excellent physical and chemical properties [1,2]. Especially in the field of microelectronics, perovskite ceramic materials with a general chemical formula ABO_3_ have been extensively used in microcircuit, modules and devices [3]. LaTiO_3_ and SrTiO_3_ both are ferroelectric ceramic and it has been reported that they have good dielectric properties, such as high dielectric constant, low dielectric loss and good temperature coefficient, *etc.* In addition, in recent years, lanthanum-doped and strontium-doped ceramic composite material as a new kind of perovskite ceramic material has been widely researched and used as solid fuel cell anode material, microwave dielectric material and photo-dew-sensitive material, *etc.* [4,5,6,7,8]. Therefore, it could be supposed that La_1__−*x*_Sr*_x_*TiO_3+δ_ (LST) may have better properties and potential applications. However, the dielectric and photoelectric properties of bulk LST are currently rarely reported.

The main purpose of this paper is to do a deep research of the dielectric and photoelectric properties of LST. Our previous theoretical simulated work has suggested that La_0.9_Sr_0.1_TiO_3+δ_ has higher dielectric function and refractive index [9]. Our following experimental research has confirmed that La_0.9_Sr_0.1_TiO_3+δ_ has the highest reflectivity (99% at 10.6 μm) [10]. So this paper will focus on LST with *x* = 0.1, considering the relationship between optical and electrical properties. LST was prepared by high-temperature solid state reaction method. Its phase structure was characterized and its dielectric and photoelectric properties were investigated.

## 2. Experiments

### 2.1. Sample Preparation

The LST samples were prepared by high temperature solid state reaction method using high-purity raw materials: La_2_O_3_ (99.99%), SrCO_3_ (AR) and TiO_2_ (AR). These raw materials were weighed stoichiometricly and mixed mechanically, then presintered at 1200 °C for 2 h in order to reduce the thermal stress during sintering. The presintered powders were mechanically pre-pressed into cylindrical pellets (10 mm in diameter and 1–2 mm in thickness) at 8 MPa for 5 min and then pressed using cold isostatic press at 200 MPa for 5 min. The pellet samples were finally sintered at 1550 °C for 8 h in air and followed by furnace cooling. Then we got the white samples. To study the dielectric properties of LST, both the top and bottom surfaces of the sample were coated with platinum electrodes by vacuum sputtering, and then annealed at 150 °C for 20 min to reduce the influence of contact resistance during the electric measurements.

### 2.2. Characterization

The phase composition of LST was characterized by X-ray diffraction (PANalytical Inc., X’Pert PRO MPD, Almelo, The Netherlands) with Cu Kα radiation and analyzed by JADE (version 5.0, Materials Data Inc., Livermore, CA, USA). The surface microstructure of LST was observes by scanning electron microscope (HITACHI S4800, Tokyo, Japan). The dielectric and other related electrical parameters were obtained by broadband dielectric spectroscopy and AC (alternating current) impedance spectroscopy instrument (Novocontrol Technologies Inc., Alpha-A, Montabaur, Germany) from the samples coated with platinum electrodes. This impedance spectroscopy instrument performs in a wide frequency range (0.01 Hz–6 MHz) at different temperature (room temperature ~700 °C). The photoelectric property of LST was measured at room temperature by photoconductive measurement instrument (Keithley Instruments Inc., 4200-SCS, Cleveland, OH, USA) with bare sample.

## 3. Result and Discussion

### 3.1. Phase Structure and Surface Microstructure of LST

**Figure 1:**
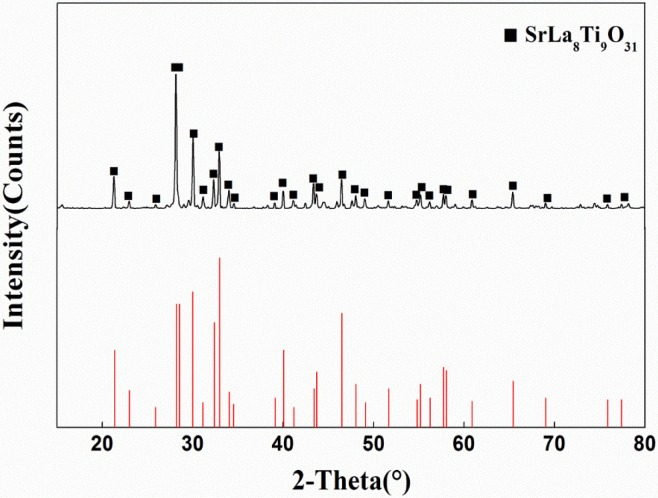
XRD pattern of La_0.9_Sr_0.1_TiO_3+*δ*_.

Figure 1 shows the XRD pattern of LST. The diffraction peaks are attributed to a single layered perovskite SrLa_8_Ti_9_O_31_ phase. It indicates that after sintering at 1650 °C for 8 h the raw materials and their intermediate materials could fully react and produce SrLa_8_Ti_9_O_31_ single phase. This experimental condition is in accord with Takamura *et al.*’s research, which obtained SrLa_8_T_i9_O_31_ single phase by the Pechiniprocess [11]. By analyzing with Jade we get the lattice parameters are: *a* = 7.810 Å, *b* = 5.533 Å, *c* = 57.010 Å, α = β = γ = 90°, which is consistent with the literatures [12].

**Figure 2:**
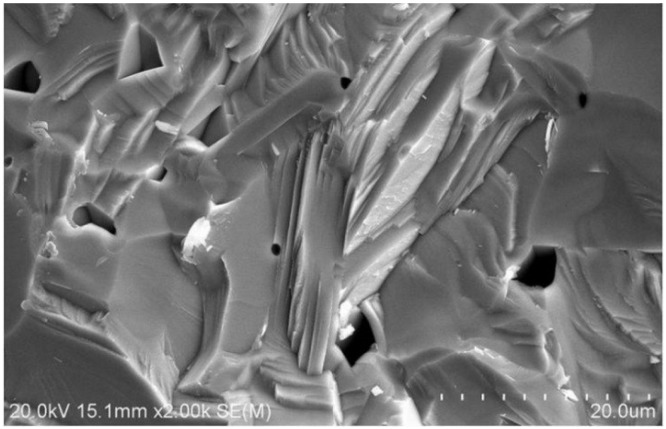
Surface microstructure of La_0.9_Sr_0.1_TiO_3+δ_ by SEM.

Figure 2 shows the surface microstructure of LST. There are little visible pores, which make the density of LST is just 4.63 g/cm^3^. Moreover, the low porosity of the sample has the benefit of improving its dielectric properties. Banno optimized [13] the formula between porosity and dielectric properties of the material, which indicates that lower porosity and less grain boundary make higher dielectric constant. Therefore, with our previous work [9], we think the dielectric properties of LST, which have layered perovskite structure and higher density, may be improved.

### 3.2. Dielectric Properties

**Figure 3:**
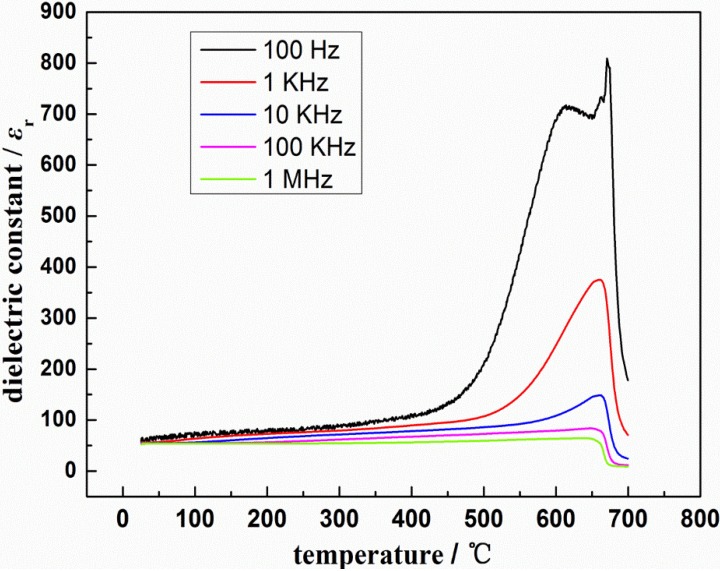
Temperature dependent ε_r_ at different frequencies of La_0.9_Sr_0.1_TiO_3+δ_.

**Figure 4:**
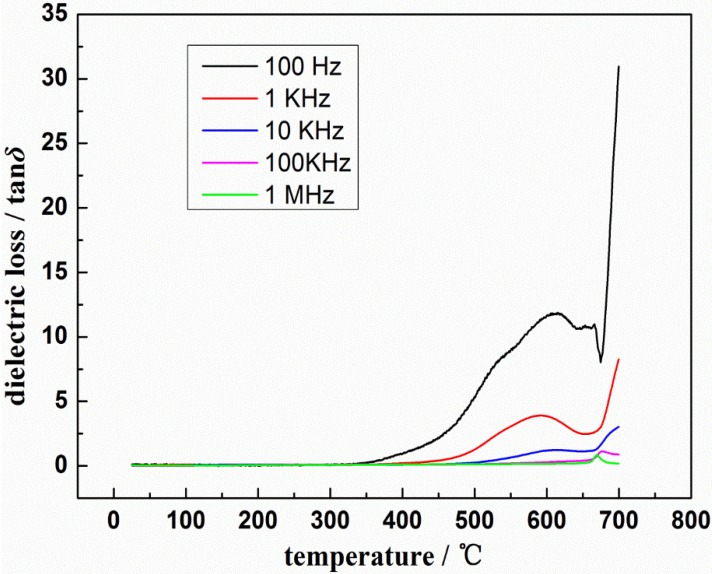
Temperature dependent tanδ at different frequencies of La_0.9_Sr_0.1_TiO_3+δ_.

The relative dielectric constant (ε_r_) and dielectric loss (tanδ) of LST as functions of temperature at different frequencies, obtained from broadband dielectric spectroscopy, are shown in Figure 3 and Figure 4. It can be observed that both relative dielectric constant and dielectric loss decrease with increasing frequency, which is a general feature of dielectric materials [14]. The relative dielectric constant and dielectric loss of this material are stable under 350 °C, especially at high frequency, in which ε_r_ is between 52 and 88 and tanδ can reach 6.5 × 10^−3^. The dielectric properties of LST are improved compared to the reported dielectric constant value of SrTiO_3_ (about 10 at room temperature) [15].

As shown in Figure 3, ε_r_ decreases with increasing frequency, because at the higher frequency lower polarization can be established. ε_r_ is almost a constant under 500 °C, which may be due to lower relaxation polarization. A similar phenomenon has been observed in Pati’s research [16]. In the high-temperature region (500–650 °C), there is a sharp increase in ε_r_. Its higher value may be due to space charge polarization which comes from mobility of ions and defects in the material, which is similar to Abhijit’s report [17]. However, in the much higher temperature range (650–700 °C), high temperature makes the heat stimulation more strongly, which causes the ferroelectric domain wall motion become more difficult. Therefore, the polarization state of disorder has become the major influence factor of ε_r_ and this fact leads to a sharp decrease in ε_r_ with increasing temperature, especially for low frequencies [18]. *Tc* of LST is thus located in the temperature region of 500–650 °C.

As shown in Figure 4, the value of tanδ increases with increasing temperature (room temperature ~600 °C). It’s associated to ionic conductivity, which is caused by the loss of oxygen during high-temperature sintering. Thus, this defect plays an important role in the conductivity of LST, which causes the dielectric loss increase [19]. Between 600 and about 680 °C, tanδ slightly decreases with increasing temperature, which is due to the decreasing of the relaxation time [16,20]. At higher temperature (about 680–700 °C), a sharp increase in tanδ is resulted by the scattering of thermally activated charge carriers [21] and the presence of some defects such as oxygen vacancies in the sample. In this temperature region, the conductivity becomes the main reason of rise in tanδ. still does not exist in the ferroelectric domain’s wall may be another reason [20] as well. Besides, the dielectric loss decreased with the increasing in frequency. It can be explained by the following relationship among dielectric loss, conductivity and frequency [22]:

(1) $$\text{tanδ}\propto \frac{\sigma }{\omega }$$

where tanδ is the dielectric loss, σ is the conductivity and ω is the frequency. The increase in the conductivity is smaller than that of frequency. Therefore, the dielectric loss decreased with the increasing in frequency in the whole temperature range.

### 3.3. AC Impedance Spectra Analysis

**Figure 5:**
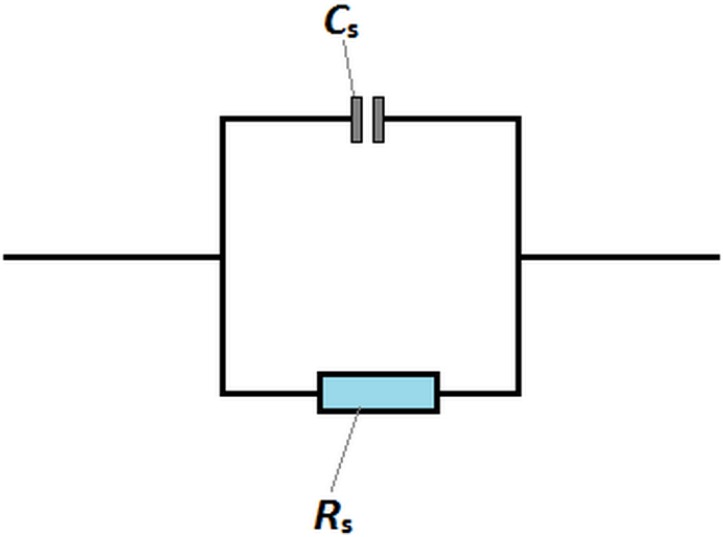
Complex impedance spectrum equivalent electrical circuit.

The AC complex impedance spectroscopy is a widely used nondestructive technique to investigate the electrical process and conductive mechanism of materials. This technique is very effective to study the contribution of impedance and other related parameters along with the equivalent circuits. In this work, the LST sample coated with platinum electrodes can be regarded as a simple capacitor, the equivalent circuit of which is shown in Figure 5 [23]. Moreover, the equivalent circuit can be summed from a single *RC* (resistance and capacitance) circuit with parallel combination. So its real part (resistive, *Z*_s_’) and imaginary part (reactive, *Z*_s_”) of complex impedance (*Z*_s_*) can be calculated using the following basic equation [24]:

(2) $${Z_{s}}^{*}=Z_{s}’-jZ_{s}”=R_{s}-(j/\omega C_{s})$$

where ω is the angular frequency, *R*_s_ and *C*_s_ are resistance and capacitance respectively.

**Figure 6:**
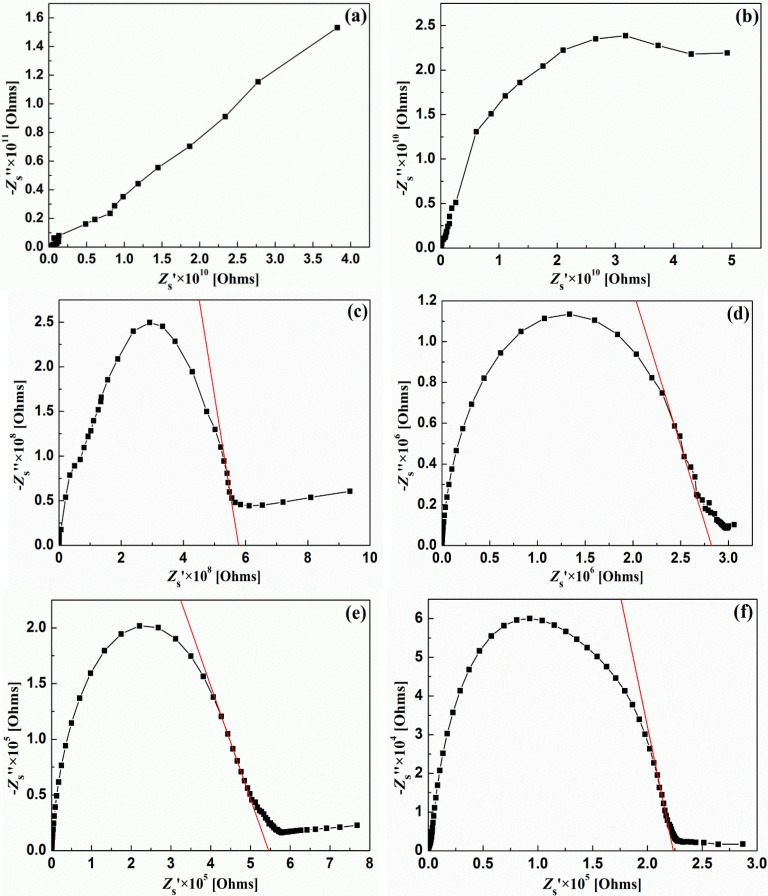
Complex impedance spectra of La_0.9_Sr_0.1_TiO_3+δ_: (**a**) 100 °C; (**b**) 200 °C; (**c**) 300 °C; (**d**) 400 °C; (**e**) 500 °C; (**f**) 600 °C.

Figure 6 shows the complex impedance spectra of LST at different temperature (100–600 °C) over a wide range of frequency (0.01 Hz–6 MHz). The impedance of this material is characterized by the formation of semicircular arcs which can mainly be attributed to a parallel combination of resistance and capacitance. According to the equivalent circuit of the sample, the extent of intercept of semicircles on the real axis (*Z*_s_’) provides the value of resistance, which could be used to calculate the conductivity of the sample. But generally, the crossover point between semicircular arcs and the real axis at low frequency may not exist, as shown in Figure 6a,b, which is because the intrinsic resistance of this material at low temperature is very large and the crossover point is outside of the testing scope. As shown in Figure 6c–f, because of some non-ideal effects [25], the crossover point at low frequency still does not exist, so it should be tangent to get the crossover point. It can be seen that as temperature increases from 100 °C, the arcs gradually becomes semicircular and the extent of intercept of semicircle on the real axis decreases which means the resistance of the sample decreases with increasing temperature. The exact calculated values of resistance (*R*_s_) and conductivity (σ_s_) at different temperature, from the tangent curves, are listed in Table 1, which is similar to Hashimoto’s research [26]. The range of resistance is from 573 MΩ (300 °C) to 228 KΩ (600 °C), and compared with traditional perovskite ceramic, like LaTiO_3_, its resistance was low. This obvious decreasing of resistance is due to the increasing of thermally activated charge carriers and the existence of some additional bands in the sample [27].

**Table 1:** Comparison of impedance parameters’ at different temperature.

Temperature (°C)	*R*_s_ (Ω)	σ_s_ (S/m)
300	573 M	9.249 × 10^−^^9^
400	2.84 M	1.866 × 10^−^^6^
500	550 K	9.635 × 10^−^^6^
600	228 K	2.325 × 10^−^^5^

### 3.4. Photoelectric Property

**Figure 7:**
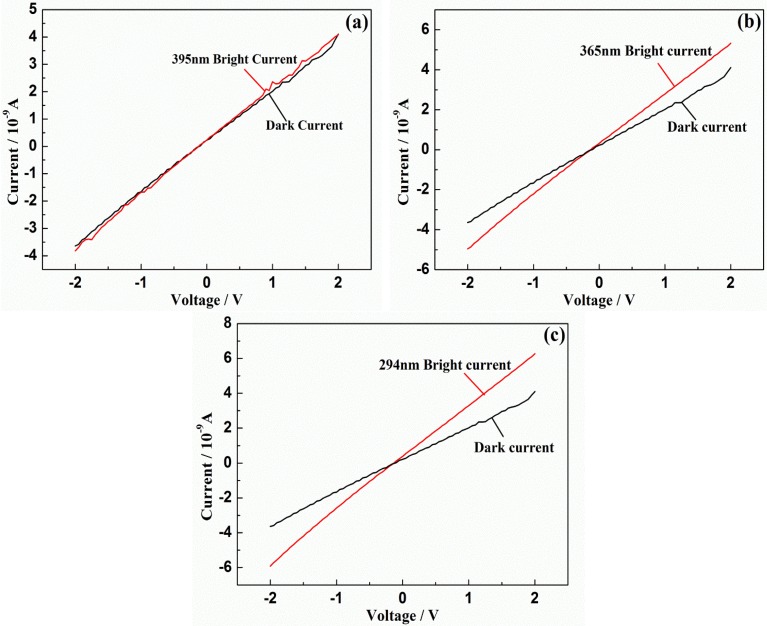
Photoconductivity instrument of La_0.9_Sr_0.1_TiO_3+δ_: (**a**) 395 nm; (**b**) 365 nm; (**c**) 294 nm.

To study its photoelectric properties is significant for us to understand more about this material and then to lay a foundation for it to apply in some related fields. In this work, the performance of LST in ultraviolet light is tested by photoconductive measurement. Figure 7 shows the bright current (red line) under ultraviolet light illumination (395 nm, 365 nm, 294 nm) and the dark current (black line) as a background, but no light, as functions of applied voltage in the range of −2.0–2.0 V. In Figure 7a, when the wavelength of the incoming light is 395 nm, the curves of the bright current and dark current are very close. This is because the photon’s energy of 395 nm wavelength light is not enough to significantly stimulate the valence band electron. In Figure 7b,c under the light wavelength of 365 nm and 294 nm, compared with the dark current the bright current increased by 50% at most. It is indicated that the photon’s energy of 365 nm and 294 nm wavelength lights can stimulate a large number of valence band electron. So the increasing of carries density of LST leads to an obvious increasing of bright current. The electron of 365 nm light has less energy and it is closer to band gap *E*_g_ of LST. The result of photoconductive measurement under ultraviolet light illumination suggests that 365 nm electron already can stimulate a large number of valence band electron of LST. The band gap *E*_g_ of LST can be calculated based on this photon energy and *E*_g_ is about 3.39 eV. In addition, the band gap of LST also has been evaluated from the activation energy based on the temperature dependence of resistivity, which is about 2.6 eV, but the activation energy may be reduced by the defects [28], so the *E*_g_ of 3.39 eV we calculated is acceptable. The value of *E*_g_ indicates that La_0.9_Sr_0.1_TiO_3+δ_ is a kind of wide-band-gap semiconductor materials [29], as the typical semiconductor material SrTiO_3_ [30]. Wide-band-gap semiconductor materials as a kind of electronic material can have a good anti-interference ability [31]. Although LST is Mott insulator, it does not satisfy the transition conditions in our study [32,33], so LST still shows insulating properties.

## 4. Conclusions

The bulk material of La_0.9_Sr_0.1_TiO_3+δ_ ceramic with perovskite structure was prepared by high-temperature solid-state reaction method. After sintering at 1650 °C for 8 h, the SrLa_8_Ti_9_O_31_ single phase was obtained. Its relative dielectric constant and dielectric loss of are stable under 350 °C especially at high frequency, well the relative dielectric constant is between 52 and 88 and dielectric loss can reach 6.5 × 10^−3^. The value of conductivity was increased with rise in temperature, which suggests the existence of negative temperature coefficient of the resistance in this material. The conductivity of La_0.9_Sr_0.1_TiO_3+δ_ can reach 2.325 × 10^−5^ at most. The calculated band gap (3.39 eV) of this material indicates that this kind of material belongs to wide-band-gap semiconductor materials. Its dielectric properties and forbidden bandwidth indicate it could be an electronic material.
